# Varied utilisation of health provision by Arab and Jewish residents in Israel

**DOI:** 10.1186/s12939-015-0193-8

**Published:** 2015-08-07

**Authors:** Jo Southern, Hector Roizin, Muhannad Daana, Carmit Rubin, Samantha Hasleton, Adi Cohen, Aviva Goral, Galia Rahav, Meir Raz, Gili Regev-Yochay, for the PICR group

**Affiliations:** Immunisation Department, Health Protection, Public Health England, 61 Colindale Ave, London, NW9 5EQ UK; Jerusalem-Hashfela District, Macabbi Healthcare Services, Modiin, Israel; Infectious Disease Epidemiology Section, The Gertner Institute, Tel Hashomer, 52621 Israel; Infectious Disease Unit, Chaim Sheba Medical Center, Ramat Gan, 52621 Israel

## Abstract

**Introduction:**

Provision of healthcare is considered a basic human right. Delivery and uptake is affected by many complex factors. Routine vaccinations are provided free of charge in Israel to all residents. The Palestinian Israeli Collaborative Research (PICR) group conducted research on vaccine impact at eight primary care facilities in east Jerusalem (EJ) and central Israel (IL) which allowed assessment and comparison of interactions of these Arab and Jewish populations, respectively, with healthcare services.

**Methods:**

Families attending clinic with a child under five years old were invited to participate. Utilisation of healthcare was assessed using data from standardise questionnaires completed after enrolment, using proxies of vaccination status, antibiotic use, primary care physician and hospital visits as well as demographics such as household size. Differences between EJ and IL were assessed using chi squared tests; univariate analyses identified potential confounders which were tested in a multiple logistic regression model for any independent associations between region and outcome.

**Results:**

Children in EJ were significantly more likely to live in larger households, with tobacco smokers, to have been breastfed, hospitalised and used antibiotics recently than those in IL, who were significantly more likely to have recently seen a primary care physician (all p < 0.01). Receipt of routine vaccinations, given at well baby clinics, was similar between the regions at above 95 % (p = 0.11), except for influenza which was delivered separately at primary physician clinics to 5 % (EJ) and 12 % (IL). Receipt of pneumococcal vaccine when paid for separately was significantly higher in IL than EJ (3 % vs 31 %). Multivariate analysis identified the most important independent predictors of these differences as region, age and household size.

**Conclusions:**

Healthcare in Israel is of a very high standard, but it is not uniformly utilised within the community in all geographical areas, though in some key areas, such as uptake of most routine childhood vaccination, equality seems to be achieved. To ensure excellent healthcare is achieved across the population, inequalities must be addressed, for instance in health promotion and other activities, which could improve and normalise health outcomes.

## Introduction

Healthcare is considered a basic human right [1, 2], with access to medical care essential in establishing and maintaining efficiently functioning communities.

Access to and utilisation of health care provisions at both the individual and population level is complex. Many factors are involved in how populations access health care including financial, organisational, social and cultural variability, meaning affordability, physical access, and acceptability of the service, not only its supply. The availability of services and barriers to access have to be considered in the context of differing perspectives, health needs and material and cultural settings of diverse groups in society [3].

The opportunity to assess the utilisation of healthcare facilities in discrete communities served under the same health policy was provided through a study conducted in primary care facilities by the Palestinian Israeli Collaborative Research group (PICR) [4, 5]. While the major aim of the PICR study was to assess the impact of pneumococcal vaccination, data were also recorded on how these communities accessed medical care. In this analysis, utilisation of healthcare provision was assessed using the proxies of visits to the primary pediatrician, prescription of antibiotics, receipt of vaccines (primary, influenza as well as pneumococcal conjugate vaccine (PCV7)) and hospitalisations. As with many countries, provision of vaccines in Israel is under national policy guidance from the Health Ministry, which delivers a vaccination programme across all age groups free of charge, known as the National Immunisation Policy (NIP) [6]. The programme is delivered in primary care under the “Uniform Benefits Package”, which is managed by four non-profit Health Maintenance Organisations (HMOs), that are supported by employer contributions and the National Insurance Institute, to which registration with one is compulsory for all citizens and residents by law [7]. It is of note that primary immunisations in the NIP are delivered in Israel at specialised “well baby” clinics, where babies are also taken for routine check-ups and weighing etc., but that influenza vaccines are delivered through the primary care physician service.

PCV7 was approved for use in 2007 in Israel, and introduced into the Israeli NIP in July 2009, under a 2, 4, 12 month schedule, with a two dose catch-up for those under two years of age, free of charge.

## Method

### Institutional review board (IRB) and patient consent

IRB approvals were given by local committees of the Sheba Medical Centre and Macabbi Healthcare Services (MHS). Written informed consent was given by a parent for each participating child before enrolment.

### Districts, clinics and study populations

Eight clinics in two districts participated: 1) East Jerusalem (EJ) District - four large MHS clinics located in different neighbourhoods in East Jerusalem, serving the exclusively Palestinian population living there, who have been considered Israeli residents since 1967 and thus treated according to Israeli Health law and policy. 2) Central Israel (IL) District - four similarly large MHS clinics, located in three cities in Hashfela District, Bat-Yam, Rishon Lezion, and Holon, serving the population living there (>99 % Jewish population).

Any child under five years of age, who visited any of the participating clinics for any reason in the two districts (EJ and IL) during May-July of two consecutive years (2009–2010), was recruited if a parent agreed and gave written informed consent. All participants were managed according to the Israeli Health policy, insured by the same HMO; MHS, one of the major HMOs in Israel, treating approximately one third of the Israeli population.

### Screening

Information from medical files recorded as part of the study documentation included the diagnosis, if any, at the screening visit as well as medical and vaccination histories since birth. In addition, trained study coordinators interviewed parents using standardised questionnaires to collect demographic data and information on antibiotic use that may have not been recorded in the medical files. Where any data differed between these two sources, for instance for vaccines administered, the information recorded in the primary care pediatrician notes was considered correct. Data recorded included information relating to health outcomes as indicators of engagement with health services, including: physician visits, antibiotic prescriptions and hospitalisations both current and in the previous three months; receipt of influenza, PCV7 vaccines. Data relating to confounders that were included in the current analyses were: age; gender; family size and specifically number of siblings; parent(s) smoking tobacco in the household; diagnosed comorbidities.

### Statistical analyses

Descriptive statistics are given as frequency distributions for each of the outcomes and confounders within region (EJ and IL). The chi-square test was used to assess differences between regions. Data on potential confounders were also available from the study questionnaires and were included in the analyses here. Variables that were shown to be significant in the univariate analysis (p < 0.05) were all included in multiple logistic regression analyses, to assess whether there was an independent association between region and outcome after controlling for all other factors, as well as to determine the independent predictors for vaccination compliance. All statistical analyses were performed using SAS 9.4.

## Results

### Lifestyle factors (Table 1)

**Table 1:** Demographics of the two populations under Israeli health policy included in the study, East Jerusalem (EJ) and Israel (IL)

Variable	EJ	IL	P-value
N	659	1099	N/a
Age of child (years), mean, sd	2.04, 1.42	1.95, 1.35	0.27
Percentage males	57.8	57.3	0.84
Mean number of people in household	4.9	4.4	<0.01
Proportions with:			
2-3 people	24.1	23.6	
4-6 people	57.7	71.1	
7+ people	18.2	5.3	
Mean number of physician visits per child in previous three months	2.9	4.0	<0.01
Proportion of children with in-patient hospital episodes in previous three months	11.5	7.2	<0.01
Proportion of children ever breastfed	94.6	63.3	<0.01
Proportion of children breastfed for more than 6 months	55.6	18.3	<0.01
Proportion of children living in a house where tobacco is smoked	49.47	26.23	<0.01
Proportion with male smoker in house	45.4	28.9	0.0125
Proportion with female smoker in house	8.7	21.6	<0.01
Among children with a diagnosis, proportion of:			
URTI^a^	66.0	49.0	<0.01
Other infections^b^	17.6	30.0	0.0011
Other non-infectious issues^c^	16.5	21.3	0.16
The proportion of prescribed antibiotics among those with an infectious diagnosis on screening day	36.4	12.7	<0.01
Proportion of children who received antibiotics in the previous three months	28.2	11.9	<0.01
Proportion of children with complete primary immunisation, according to the NIP	98.9	97.9	0.11
Proportion of children who had ever received influenza vaccine ^	4.7	11.8	<0.01
Proportion of children who received PCV7 vaccine in 2009^^	2.6	30.7	<0.01
Proportion of children who received PCV7 vaccine in 2010^^^	55.6	62.5	0.04

^a^URTI included pneumonia, URTI, otitis media, tonsillitis

^b^Other infections included UTI, gastroenteritis, skin infection, viral infection

^c^Other non-infectious issues included non-infectious GI and respiratory problems, surgery, treatment of wound/ injury, jaundice, anaemia

^Influenza vaccine was included in the NIP free of charge and delivered by primary physicians from 2006

Before PCV7 (Prevenar) was included in the NIP free of charge (since July2009)

^^^One year after PCV7 was included in the NIP

Analyses demonstrated the inclusion of a similar proportion of index children in terms of age and sex (p = 0.19, and p = 0.84 respectively). However, it was apparent that these children lived in significantly different size of households with the majority in both locations living in households of 4–6 people but a higher proportion in EJ in households of seven or more people (18.2 % vs. 5.3 %, p < 0.01). Significantly more children in EJ lived in a house where tobacco was smoked (49.5 % vs. 26.2 %, p < 0.01) and where that smoker was more commonly male than female (45.4 % vs 8.7 %, p < 0.01)) than in IL (28.9 % vs 21.6 %, p = 0.06). Breastfeeding was provided to the majority of babies in EJ compared to IL (94.6 % vs.63.3 %; p < 0.01) and the proportion breastfed for at least six months was significantly higher in EJ compared to IL (55.6 % vs. 18.3 %; p < 0.01).

### Interaction with primary care physician and hospital

Children in IL were more likely to have seen a primary care physician in the three months prior to the current visit than those in EJ (94.2 % vs. 82.3 %; p < 0.01), who were conversely more likely to have been hospitalised within the same period (11.5 % vs. 7.2 %; p < 0.01). The physician diagnosis at the screening visit differed between the groups and was most likely to be a URTI in EJ (65.9 % vs 49.0 % in IL; p < 0.01). In IL there were significantly higher proportions of visits for other infections as well as non-infectious issues (51.0 % vs. 34.1 %; p < 0.01). Interestingly the prescription of antibiotics at the screening visit was significantly higher in EJ than IL (36.4 % vs 12.7 %, p < 0.01), with a similar pattern of families reporting antibiotic use for the child in the preceding three months (28.2 % in EJ vs 11.9 % in IL, p < 0.01). While use of most antibiotic groups was somewhat higher in EJ, this was particularly significant for macrolides at 29.7 % of antibiotics used in EJ vs. 15.4 % of those used in Il, (p < 0.0001). The source of these prior medications was not known i.e., whether prescribed by the physician or bought over the counter in a pharmacy, as is possible in some areas. This pattern was maintained even when taking into consideration information from the physician notes regarding antibiotic use as well as information from the parent (51.6 % vs 28.7 %; p < 0.01).

### Vaccinations

Completion of primary immunisations delivered at the well baby clinics was similar, at above 95 % for both regions (p = 0.11). Similarly, within one year of inclusion of PVC7 in the NIP in 2010, similar proportions of children were vaccinated in the two regions; 55.6 % of the EJ group and 62.5 % of the IL group (adjusted OR 0.783, 95 % CI: 0.54-1.14, p = 0.20), of which 60.3 % and 46.7 % respectively had completed vaccination by six months of age (p = 0.12).

It is interesting to note that when PCV7 was available to purchase in 2009, prior to its inclusion in the NIP, 2.6 % of the EJ group and 30.7 % of the IL group were vaccinated (p < 0.01). The multivariate models (Tables 2 & 3) indeed show that region was a significant independent predictor of vaccination compliance before PCV was included in the NIP, when purchasing PCV was associated with high costs (aOR = 0.064, 95 % CI: 0.031-0.131). Yet, after its inclusion in the NIP, compliance with PCV vaccination was relatively high and not significantly different between the two regions (aOR = 0.783, 95 % CI: 0.539-1.135). Other independent predictors for lower PCV immunisation rates were: larger households (>3 people) (either before introduction to NIP aOR = 0.527, 95 % CI 0.342-0.813 or after inclusion in NIP aOR = 0.485, 95 % CI 0.267-0.879), a parent being a smoker (predicted lower vaccination rate in the first year aOR = 0.519, 95 % CI 0.329-0.818 but not in the second year). Predictors for higher PCV immunisation rates were age, between six months and two years, compared to those above two years of age, in both survey years (aOR = 3.885, 95 % CI 2.521-5.988 in the first year and aOR = 8.754 95 % CI 6.157-12.448 in the second year) and multiple visits to the clinic in the second year (aOR = 2.312, 95 % CI 1.066-5.011).

**Table 2:** Results of the multivariate analysis for predictors of PCV7 vaccination

a. Before introduction to the NIP (2009)					
Predictors		OR	CI		P value
Region	IL	Ref			
	EJ	0.064	0.031	0.131	<.0001
Age					<.0001
	>2 years	Ref			
	6-24 m	2.353	1.271	4.356	0.0065
	<6 months	3.885	2.521	5.988	<.0001
Tabacco smoking in the household	No	Ref			
	Yes	0.519	0.329	0.818	0.0047
Number of household members	2-3	Ref			0.0031
	4-6	0.527	0.342	0.813	0.0038
	7+	0.263	0.093	0.741	0.0115
Breastfeeding	No	Ref			
	Yes	1.186	0.784	1.794	0.4186
Number of physician visits during the previous 3 m	0	Ref			
	1+	1.606	0.826	3.122	0.1624
b: After introduction of PCV7 into NIP (2010)					
Predictors		OR	CI		P value
Region	IL	Ref			
	EJ	0.783	0.539	1.135	0.197
Age					<.0001
	>2 years	Ref			
	6-24 m	8.75	6.16	12.45	<.0001
	<6 months	1.82	1.2	2.75	0.0045
Tabacco smoking in the household	No	Ref			
	Yes	1.03	0.73	1.46	0.8562
Number of household members	2-3	Ref			0.0031
	4 - 6	0.64	0.44	0.93	0.021
	7+	0.49	0.21	0.88	0.0172
Breastfeeding	No	Ref			
	Yes	1.078	0.73	1.59	0.7026
Number of physician visits during the previous 3 m	0	Ref			
	1+	2.31	1.01	5.01	0.0338

**Table 3:** Results of the multivariate analysis for predictors of flu vaccination

Predictors		OR	CI		P value
Region	IL	Ref			
	EJ	0.357	0.226	0.563	<.0001
Age	>2 years	Ref			
	6 m – 24 m	0.525	0.369	0.747	0.0003
Tabacco smoking in the household	No	Ref			
	Yes	0.715	0.487	1.052	0.0886
Number of household members	2 - 3	Ref			0.0154
	4 - 6	0.566	0.384	0.834	0.004
	7+	0.612	0.306	1.227	0.1667
Breastfeeding	No	Ref			
	Yes	1.316	0.887	1.952	0.1725
Number of physician visits during the previous 3 m	0	Ref			
	1+	1.427	0.699	2.912	0.3291

Despite its inclusion in the NIP, only a minority of children were vaccinated against influenza in both regions, but significantly fewer in EJ than IL (4.7 % vs 11.8 %; adjusted OR 0.357, 95 % CI: 0.23-0.56; p < 0.001). Other factors that predicted the lower influenza vaccination uptake were younger age and larger households (Table 2).

Multivariate analysis that assessed independent predictors associated with antibiotic use in the three months prior to screening, revealed that antibiotic use was higher in EJ compared to IL (AOR = 3.339, 95 % CI 2.612-4.267), and that babies younger than six months old were less likely to be treated with antibiotics compared with children older than two years (AOR = 0.152, 95 % CI 0.097-0.2375). This analysis also demonstrated that children who had visited the clinic more than once in the previous three months tended to have higher rate of antibiotic use compared to children who had not (AOR = 2.198, 95 % CI 2.612-4.267).

Multivariate analysis of independent predictors for receiving influenza vaccination (Table 3) identified region as the most important predictor (AOR = 0.357, 95 % CI: 0.226-0.563 for EJ compared to IL). Other predictors that were independently associated with influenza vaccination were age: children between six months and two years had lower immunisation rate compared to those older than two years (AOR = 0.525, 95 % CI0.369-0.747), and size of household, with lower rate of immunisation in households with 4–6 people compared to 2–3 people (AOR = 0.566 95 % CI 0.384-0.834).

## Discussion

The analyses presented here demonstrate the varied household structures and behaviours in the two regions included, as well as distinctly varied interaction with healthcare services. The reasons for these differences are not clear from the analyses performed here, but are undoubtedly complex and rooted in cultural and socioeconomic differences.

In terms of finances, all Israeli citizens and residents, i.e., all those included in this analysis, have access to basic healthcare from the State, through their HMO. This encompasses all paediatric vaccinations and attendance at well baby clinics, as well as diagnosis, consultation and medical treatment including the supply of medications (though a small percentage of the cost of some medications may be paid by the patient), hospitalisation and rehabilitation [7]. The material and cultural settings for Jews and Arabs living in Israel are certainly diverse, with varied population and family structures as documented in a 2012 report [8]. This reported that those under 15 years of age constituted 37.5 % and 25.5 % of the populations respectively; and the average family size at 4.7 people compared to 3.5, respectively, and those over 15 years of age living with their family in 98 % and 89 % of cases, respectively. There are socioeconomic strata in Israeli society, as in any country, and these are often linked through complex mechanisms to religion and ethnicity [9]. Of significant note are also the potential differences that exist between Arab residents and Arab citizens of Israel. Most previous analyses [12, 13] have considered them as one group but the residents (who have held this status only since 1967), as per the EJ group in the current study, are usually considered of lower socioeconomic status than the citizens (who have held the status since Israel’s establishment in 1948), and this report may be the first to assess them as a separate group in the considerations presented here.

National datasets illustrate that health disparities exist between Jews and Arabs living in Israel, which are of course multifactorial, but nevertheless are clear. For example, a report from the Ministry for Health [10], documents proxies for health inequalities including: disparities in infant mortality, at 3 per 1000 live births for Jews and 7 per 1000 for Arabs; a gap in life expectancy with Jews living longer, which increased from two years in 1998 to 3.7 years in 2008. In context, the WHO reported infant mortality rates for 2011 in Israel’s neighbouring countries as 18 per 100,000 for both Jordan and Egypt compared to 4 per 100,000 for Israel [11].

Some behavioural factors also varied between the populations, for instance in men over 21 years of age, smoking was reported by 44 % of Arabs compared to 24 % of Jews. Indeed, our study demonstrated that in 49.5 % of Arab households, smoking was reported, compared to 26 % of Jewish households. Interestingly, a higher proportion of women smoked in the Jewish population (22 % compared to 9 % Arab women).

In 2009 the Sikkuy organisation, which is the “Association for the Advancement of Civic Equality”, issued a report [12] documenting equality between Jewish and Arab citizens of Israel according to various indices. In the area of health, they concluded there were inequalities in both the infrastructure and process relating to healthcare between Arabs and Jews, as illustrated by the current analysis, which were widening over time.

Self referral to health services follows different patterns in different populations, often according to social norms as well as the availability of such services. For example, as opposed to west Jerusalem, in IL, where there are 24 h clinics for specialist emergency pediatric care, in EJ there is no such service. Use of the different services may therefore be related to accessibility rather than severity of illness. This potentially contributes to motivators for the health seeking patterns observed in the current study, which also reflect those elicited in a cross-sectional telephone survey conducted in Israel [13]. Self perception of health, which was demonstrated as better in Arabs living in Israel, who reported less chronic diseases than Jews [14] may also contribute to the frequency of and nature of interactions with health services.

The higher antibiotic use observed in EJ compared to IL, could be a result of various factors that were not assessed in this study. While all primary care physicians in the study worked for the same HMO, the education and training of these physicians may be different (different experience and proportion of board certified physicians). Furthermore, antibiotic use could be different due to potential over the counter use which is not fully enforced in EJ, while totally unavailable in IL, many other factors determine antibiotic prescription and use and were beyond the scope of this study."

A WHO report documented that, with regard to vaccine coverage, Israel was eighth of 23 western European countries, with 93 % coverage for measles immunisation as an indicator [15]. Of note is the provision of training to healthcare professionals, which inevitably impacts on delivery of care and engagement with the community. Previously, most documentation including training materials and publicity, was provided in Hebrew, though this is now encouragingly changing to encompass languages of local populations. Interestingly, this study illustrates that while compliance to routine childhood vaccinations in the NIP, such as MMR, hepatitis, DTP etc., was similarly high in both populations (p = 0.11), compliance to influenza vaccination, which is also included in the NIP was low, (IL 11.8 %, EJ 4.7 %) and significantly lower in EJ (p < 0.01). A major difference in the delivery of these vaccines exists in that all childhood vaccines except influenza are given at well baby clinics, where activities such as weighing and other routine visits are also undertaken. Influenza vaccination is given by primary care physicians so necessitating a specifically booked and separately attended appointment. Such implementation factors may impact vaccine uptake, as illustrated in this analysis where there was a huge disparity in the proportion of children vaccinated against influenza compared to all other routine infant immunisations, despite their seemingly similar provision through HMOs and being provided free of charge. In demonstrating the complexities of the underlying issues for differences in uptake, the even wider gap in PCV7 uptake before its 2010 inclusion in the NIP was probably driven mainly by financial factors, but may also have involved awareness issues in health professionals and or the public, ordering and cash flow issues in local pharmacies and medical services and in the general availability of private medical facilities in the two localities or any number of other issues which may or may not be immediately apparent.

Various studies have assessed uptake of routine immunisations in different communities. In the US, the Health Plan Employer Data and Information Set (HEDIS) was used to assess trends in childhood immunisation and factors associated with higher rates over time between 1999–2002 [16]. They concluded that significantly lower uptake rates were observed in areas with higher proportions of ethnic minorities and that uptake rates were significantly higher in areas where children visited physicians more such that they recommended such visits be seen as an opportunity to assess and address immunisation status.

A retrospective analysis of data from the 2000–2001 Canadian Community Health Survey assessed uptake of flu vaccination, as well as mammography and pap smear, in those aged ≥12 years [17]. They reported that analysing true rates of preventive health measures faced obstacles, but did note that programmes to target high risk groups were indicated to improve overall use of these services.

In Malawi, vaccine coverage among regions varied from 2 % to 74 % across geographical regions when all children 10–60 months old were included [18]. They noted that increased interaction with health services, for instance with a high percentage of deliveries attended by health personnel were also characterized by a higher coverage, as was indicators of higher socioeconomic status such as literacy, though they commented that such factors had little effect. They concluded that political intervention may be able to affect higher rates, as were achieved in some regions.

These studies give context to the current analysis such that they demonstrate the complexities both collecting and analysing data to illustrate vaccine uptake in communities, and that in Israel delivery of the routine childhood immunisation programme is achieved to a very high level across the community compared to other countries.

In conclusion, it is clear that healthcare in Israel is of a very high standard, but is not uniformly utilised within the community in all geographical areas. The reasons for this are complex and multifactorial, but it is clear that there are distinct differences according to the population groups into which individuals fall. Investigation of some of these factors will be included in future studies by this group. It is important to note that in some key areas, such as uptake of childhood vaccination, equality seems to be being achieved for those vaccinations included in the NIP and delivered through well baby clinics. However, in order to extend this equality and ensure that excellent healthcare is achieved across the population in all areas, inequalities must be addressed, for instance in health promotion and other activities, which could improve and normalise outcomes such as chronic disease management and life expectancy.
